# Palmitoleic acid as a coordinating molecule between the invasive pinewood nematode and its newly associated fungi

**DOI:** 10.1038/s41396-023-01489-8

**Published:** 2023-08-21

**Authors:** Jing Ning, Xiaoting Gu, Jiao Zhou, Hongxia Zhang, Jianghua Sun, Lilin Zhao

**Affiliations:** 1 State Key Laboratory of Integrated Management of Pest Insects and Rodents, Institute of Zoology, Chinese Academy of Sciences, Beijing, 100101 China; 2 CAS Center for Excellence in Biotic Interactions, University of Chinese Academy of Sciences, Beijing, 100049 China; 3 Hebei Basic Science Center for Biotic Interactions/College of Life Science, Institutes of Life Science and Green Development, Hebei University, Baoding, 071002 China

**Keywords:** Microbial ecology, Symbiosis

## Abstract

Symbiotic microorganisms are ubiquitous on the body surface or internal tissues of invertebrates, providing them with benefits. Developing symbiotic relationships requires synchronization of developmental stages and physical proximity of partners. Therefore, the identification of metabolites that coordinate the reproduction of symbiotic partners is essential. This study demonstrates that palmitoleic acid (C16: 1) coordinates bilateral propagation by regulating the synchronization of reproduction between the invasive pinewood nematode (PWN) and its newly associated blue-stain fungus, *Sporothrix* sp.1. When the PWN fed on *Sporothrix* sp.1, there was a significant increase in lipid metabolism gene expression and metabolite abundance. Through further investigations, it highlighted a significant enhancement in the reproduction of the PWN through direct acquisition of C16: 1, which was abundantly present in *Sporothrix* sp.1. Furthermore, the PWN biosynthesized C16: 1 through the involvement of the stearoyl-CoA 9-desaturase gene *fat-5* and its hormone nuclear receptor *nhr-80*, which was clarified to promote the egg-laying capacity of females. Moreover, it is worth noting that the production of C16: 1 was significantly higher by the associated fungus *Sporothrix* sp.1 to enhance sporulation during the spore formation phase compared to the hypha growth phase. Thus, by coordinating the fecundity and spore production, the key lipid metabolite C16: 1 facilitates the rapid and successful colonization of a mutually beneficial symbiotic relationship between the invasive PWN and the native *Sporothrix* sp.1 within the host. This finding emphasizes the significant role of metabolite sharing and its function in promoting partner synchronization within symbiotic relationships.

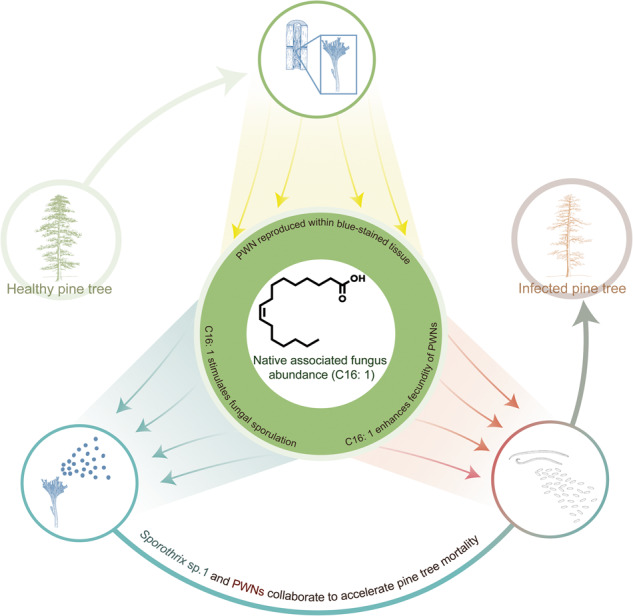

## Introduction

Symbiotic microbes are widely distributed, found not only on the body surface but also inside the gut and blood cavity of associated invertebrates. These microbes provide the partners with additional nutritional intake, growth, development, and pathogen resistance [1, 2]. Studies have reported that associated bacteria synthesize amino acids, fix nitrogen, and degrade cellulose, providing nutrition and energy to their symbiotic partners, such as ants, aphids, and cockroaches [3–6]. Some symbiotic bacteria, such as *Pseudomonas* and *Streptomyces*, can also produce polyketide toxins and antibiotics to protect their partners from pathogenic microbes and predators [7, 8]. Correspondingly, some insects have evolved a range of strategies to protect, carry, and transmit their primary associated fungi such as mycangia [9, 10]. These microbes-invertebrates interactions have formed and persisted over millions of years of evolution [11]. The establishment of a symbiotic relationship requires the developmental consistency of symbiotic partners overlapping in time and space. However, the primary focus of research has been on examining well-established symbiotic relationships, and the process of establishing new symbiotic relationships remains relatively unclear.

In fact, invasive species frequently exhibit the capacity to expeditiously establish new symbiotic associations with indigenous species, thereby assuming a conspicuous function in ameliorating the bottleneck effect encountered by small populations of the invader [1, 12]. The symbiotic microorganisms serve as suitable partners for invasive species as a beneficial factor in its successful colonization [13]. For example, some woody plants cannot be successfully introduced without forming ectomycorrhizal associations with soil fungi [14]. Conversely, invasive organisms can accelerate their establishment if they meet a new beneficial symbiotic partner in a newly invaded region [15, 16]. For instance, after invading China, the pinewood nematode (PWN), which is native to North America, quickly formed a new symbiotic partnership with the indigenous blue-stain fungus, *Sporothrix* sp.1. This enhanced the nematode’s reproductive capacity and facilitating its successful colonization in the new ecological environment [17]. Moreover, invasive organisms could further promote the spread of newly associated fungal partners in the invaded regions to occupy a major ecological niche [15, 16]. The metabolites that bilaterally coordinate the life cycles in time and space of associated partners when a new symbiotic interaction should be explored.

The entire pathogenic cycle of the PWN relies on intricate interactions with its symbiotic partners, especially symbiotic fungi [18–21]. The PWN often enters new healthy host pine trees through the young branches along with associated microorganisms, especially ophiostomatoid blue-stain fungi. Subsequently, these fungi rapidly grow and spread along the trunk of the pine tree, thereby accelerating the reproduction and migration of PWN from the tree crown to the base of the trunk. As the infestation progresses, the nutrient supply for PWN in the pine tree decreases. The spores of the ophiostomatoid fungi become the primary source of nutrition for the PWN population, and cause the xylem to turn blue [20–22]. This study focused on exploring the symbiotic invasive system between PWNs and *S*. sp. 1. We aimed to uncover the metabolites that coordinate the reproduction capacities and adaptability of both organisms. The key metabolite palmitoleic acid (C16: 1) was successfully identified through the application of genomic, transcriptomic, and metabolomic analyses, along with biochemical and molecular biology techniques. This study is a theoretical expansion of nutrient metabolites in the establishment of a new symbiotic system of the theoretical paradigm of symbiotic invasion. It also provide a framework for the study of the nutrient metabolites and biological characteristics of enhanced fertility.

## Materials and methods

### Nematode strains and fungi culture condition

**Fig. 1:**
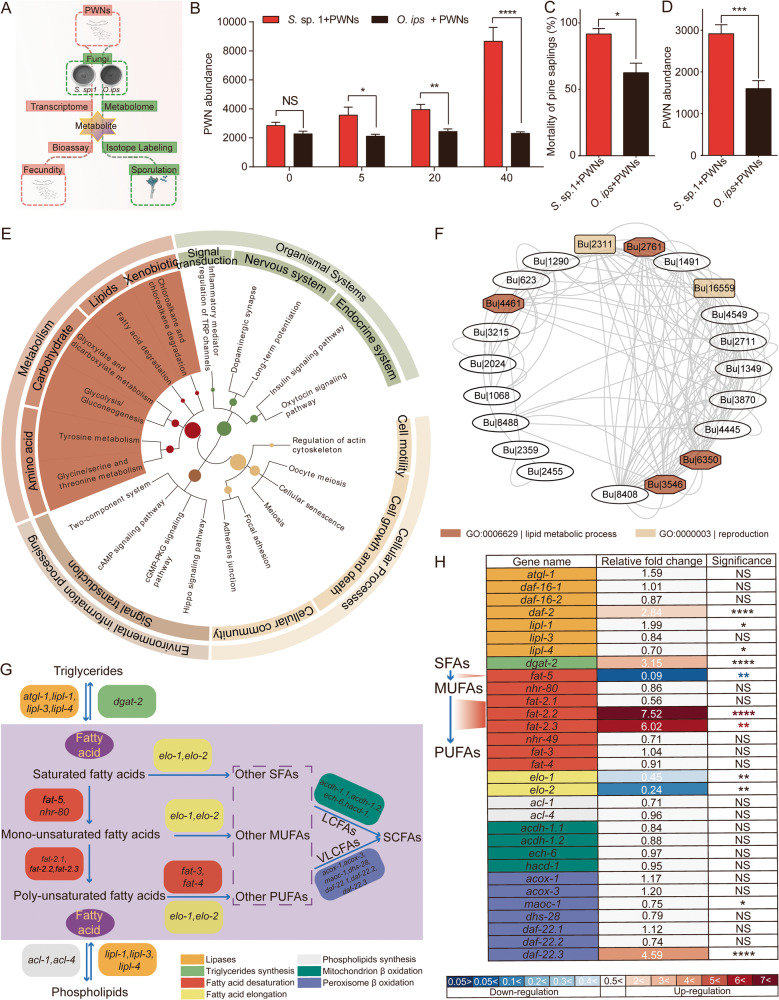
Effect of two blue-stain fungi on PWNs. **A** The experimental design diagram. **B** Abundance of CNPWN continuously cultured on two blue-stain fungi for the 0, 5th, 20th and 40th generations, and 10 nematodes were inoculated into each dish, with 10 replicates for each treatment. *, **, **** stand for significant difference under *p* < 0.05, *p* < 0.01, and *p* < 0.0001 respectively [Student’s *t* test]. **C** Mortality rate of PWNs with two blue-stain fungi on *Pinus thunbergii* was assessed, with one treatment applied to 10 trees and 3 replicates for each treatment. * stand for significant difference under *p* < 0.05 [Student’s *t* test]. **D** Population growth of PWN in *P. thunbergii*, with 10 replicates for each treatment. *** stand for significant difference under *p* < 0.001 respectively [Student’s *t* test]. **E** Distribution of KEGG function groups within up-regulated genes in PWNs continuously cultured on *Sporothrix*. sp.1 and *Ophiostoma ips* for 40 generation. **F** Co-expression network (*r* ≥ 0.8) of genes highly expressed in the *S*. sp. 1-cultured PWNs. Lines connecting genes indicate correlated expression; functional annotations: involved in reproduction (yellow filled), lipid metabolism process (orange filled). **G** Fat metabolism pathways with genes used in RT-qPCR. **H** Gene expression profiles of genes in the lipid metabolism treated with two blue-stain fungi based on RT-qPCR. [*n* = 5; *p < 0.05; **p < 0.01; ****p < 0.0001; Student’s *t* test].

The flowchart of the experimental design in this study is shown in Fig. 1A. PWN isolates from China (CNPWN) and North America (USPWN) were obtained from Zhashui, Shaanxi, China, and Pennsylvania, USA, respectively. Isolates were cultured on 2% Malt Extract Agar (MEA) with two blue-stain fungi at 25 °C. The fungi were grown on 2% MEA at 25 °C and 80% humidity for one week. *S*. sp. 1 is a local Chinese species of blue-stain fungi from the group of ophiostomatoid, while *Ophiostoma ips* is a common species in both China and North America. Voucher specimens were deposited in the culture collection (CMW) of the Forestry and Agricultural Biotechnology Institute (FABI), University of Pretoria, South Africa. Fatty acid supplementation medium, xylem powder medium were prepared and experiments were conducted following the Supplementary Methods.

### Effect of two blue-stain fungi on the fecundity of PWNs

Two species of blue-stain fungi, *S*. sp. 1 and *O. ips*, were cultured in 2% MEA. Each dish (90 mm diameter) was inoculated with 10 PWNs (5 males and 5 females) suspended in 40 μL sterile water, after the fungi had grown throughout the dish. The nematodes were then incubated at 25 °C in the dark and counted at generation 0, then after 5, 20, and 40 generations using the Baermann funnel method to measure changes in fecundity [23].

### Effect of two blue-stain fungi on the pathogenicity of PWNs

Two-to-three-year-old *Pinus thunbergii* seedlings were transplanted from a field area into pots in a greenhouse (25 °C and 40% humidity with a photoperiod of 10 L:14 D). Each of the ten pines was inoculated with approximately 1000 PWNs cultured on two blue-stain fungi, with three replicates per treatment [24]. After 20 days, the proportion of wilted black pine seedlings was recorded, and the total number of nematodes per seedling was determined by collecting nematodes from small trunk sections using the Baermann funnel method. The number of nematodes per gram of dry weight of seedling was calculated based on the dried wood chips. (*n* = 10).

### RNA-seq analyses and qRT-PCR analysis

The RNA extraction, RNA-seq analysis, cDNA synthesis, and qRT-PCR followed the protocol outlined in the Supplementary Methods. Supplementary Table S1 contains the primers used in this study. Both CNPWN and USPWN strains were continuously cultured on colonies of each of the two fungi for 40 generations. The 40th generation nematodes were prepared by combining mixed-stage nematodes. Transcriptome sequencing and qRT-PCR were performed on PWNs supplemented with C16: 1 and oleic acid (C18: 1) using the protocol described in the Supplementary Methods, with three biological replicates. The RNA-seq data of PWNs at different life-stages were obtained from the SRA database of NCBI (PRJNA798902) and analyzed using the same methods as described in the Supplementary Methods.

### Lipid droplets (LDs) staining

Lipids were visualized by staining nematodes with Oil-Red O (Sigma, Missouri, USA) and fungi with Bodipy (D3922, Molecular Probes, Carlsbad, California, USA). To ensure that the number of fungal hyphae and cells within the field of view was roughly the same, bright-field images and cell nucleus images stained with 4,6-diamidino-2-phenylindole (DAPI, Thermo Fisher Scientific, Massachusetts, USA) were captured separately. Samples were collected and washed two or three times with phosphate-buffered saline (1 × PBS) and incubated for 2 h in Oil-Red O or Bodipy solution at room temperature. After two or three washes with 1 × PBS, samples were mounted on glass slides. The nematodes were examined using differential interference contrast (DIC) microscopy (Olympus, BX51) at 10 × magnification. Fungi were examined under a Zeiss LSM 710 confocal microscope at 63× magnification with an excitation wavelength of 493 nm and an emission wavelength of 503 nm.

### Triacylglycerol (TAG) measurements

The nematodes and fungi were homogenized in 100 μL of 1 × PBS containing 0.5% Tween-20 and incubated at 70 °C for 5 min. Triglyceride reagent (Sigma) was added to the samples and they were incubated for 30 min at 37 °C. After centrifugation, the samples were transferred to 96-well plates and incubated with free glycerol reagent (Sigma) for 5 min at 37 °C. Finally, the samples were assayed using SpectraMax Plus384 at a wavelength of 540 nm.

### Determination of fatty acids (FAs) levels

To investigate the differences in FAs between two blue-stain fungi and PWNs treated by these fungi, we utilized approximately 5 mg of fungi or 10,000 nematodes. The samples were frozen in liquid nitrogen and ground in a 2% H_2_SO_4_/98% methanol solution (1 mL). Subsequently, the samples were sealed with a cap and incubated at 80 °C for 1 h. After adding 0.3 mL of hexane and 1.5 mL of H_2_O, the fatty acid methyl esters were extracted into the hexane layer by shaking and then centrifuged at 5000 × g for 10 min. We analyzed the organic phase samples using an Agilent Technologies 6890 N GC-5973N mass selective detector (GC/MS). Mass spectrometry was conducted as per the methods described in the Supplementary Methods.

### Effect of FAs on the fecundity of PWNs

Two blue-stain fungi (*S*. sp. 1 and *O. ips*) were cultured in a 2% MEA medium and we added different kinds of FAs described in the Supplementary Methods. Ten PWNs (males : females = 5 : 5) suspended in 40 μL sterile water were inoculated in the middle of each dish (12 replicates) after the fungi had grown all over the dish (60 mm diameter). The nematodes were placed in a dark incubator at 25 °C for one week, using the Baermann funnel method of separation to calculate the change in fecundity [23].

### ^31^D labeled palmitic acid (C16: 0) treatment

In order to investigate the absorption of C16: 0 and biosynthesis of C16: 1, a stable isotope of C16: 0 (^31^D-C16: 0) was utilized. *S*. sp. 1 and *O. ips* were cultured in 2% ME (20 g malt extract and 1 L deionized water) added with 1 mM ^31^D-C16: 0 and 0.1% Tergitol detergent (type NP-40, Sigma-Aldrich) for one week. PWNs were cultured on the Petri dish with *Botrytis cinerea*. Labeled fatty acid was spread evenly on the fungal mat as described in the Supplementary Methods. Collection, extraction and detection of PWNs and its FAs are described in the Supplementary Methods as well. The labeled ion are m/ z: 301 for ^31^D-C16: 0 and 297 for ^29^D-C16: 1.

### Double-stranded RNA (dsRNA) synthesis and RNAi

Double-stranded RNA synthesis was performed as previously described. Briefly, PCR was performed using the cDNA samples as templates to generate 300 bp *nhr-80* and *fat-5* specific fragments using both sense and antisense primers (Table S1) fused with T7-phage promoter sequences. Synthesis of dsRNA was accomplished by simultaneous transcription of both strands of the template by the T7 RiboMAX^TM^ Express RNAi System (Promega, USA).

The nematodes were collected and washed with Phosphate-Buffered Saline with Tween (1 × PBST) buffer. For each reaction, around 10,000 nematodes were soaked in dsRNA in a final volume of 50 μL. Control samples were soaked in dsRNA of green fluorescent protein (GFP). To dissolve the FAs in the solution, Tergitol was added as previously described. All the soaked samples were incubated in an orbital shaker at 175 rpm, 20 °C for 48 h. After soaking, the nematodes were washed three times with double distilled water. Nematodes were sorted according to sex, and experiments continued until no more eggs were laid. Twelve adults (♀: ♂ = 3: 1) were incubated in microtiter wells containing 150 μL ddH_2_O. Eggs laid were counted at room temperature after incubation at 25 °C for three days (6 replications).

### Metabolomic analysis

A comparative metabolomic analysis of *S*. sp. 1 and *O*. ips was conducted to trace the origin of FAs in PWNs. Metabolite extractions, LC-MS/MS detection, data processing, metabolite annotation and enrichment were performed as described in the Supplementary Methods. Six biological replicates were used in this study.

### Effect of fatty acid on the hypha growth, branching and sporulation of fungi

To investigate the effect of FAs on the growth and fecundity of fungi, we selected 1 mM of different kinds of FAs to assess the effect on the growth rate, dry weight, branching and sporulation of fungi. Medium preparation and experiments were performed as described in the Supplementary Methods. In addition, the FAs difference of *S*. sp. 1 between the hypha growth stage (at 3 days after inoculation) and spore formation stage (at 14 days after inoculation) was compared using GC/MS as described in the Supplementary Methods.

## Results

### *S*. sp. 1 promote the fecundity of PWNs

In this study, we investigated the effect of two blue-stain fungi, *S*. sp. 1 and *O. ips*, on the fecundity of PWNs (*S*. sp. 1 + PWNs and *O. ips* + PWNs). Nematode strains from China (CNPWNs) and North America (USPWNs) were cultured on each fungus for multiple generations. Our finding demonstrated that *S*. sp. 1 has a significant positive effect on the fecundity of PWNs compared to *O. ips*, and this effect increases with the number of generations in culture.

For CNPWNs, the PWN number per Petri dish cultured on *O. ips* in generations 0, 5, 20, and 40 were 2771, 2117, 2435, and 2310, respectively. However, the PWN number per Petri dish cultured on *S*. sp. 1 were 2850, 3572, 3950, and 8655, respectively. Statistical analysis revealed significant differences between the two groups (5th generation: df = 9.0, *t* = 2.5, *p* < *0*.05; 20^th^ generation: df= 13.4, *t* = 3.7, *p* < 0.01; 40^th^ generation: df= 9.2, *t* = 6.5, *p* < 0.0001) (Fig. 1B). Similarly, for USPWNs, the PWN number per Petri dish cultured on *O. ips* were 1815, 1810, 1995, and 2015, respectively, while for *S*. sp. 1 + PWN, the PWN number were 2550, 3050, 3300, and 6000, respectively. Statistical analysis revealed significant differences between the two groups (0th generation: df = 14.0, *t* = 2.2, *p* < 0.05; 5th generation: df = 11.7, *t* = 3.5, *p* < 0.01; 20^th^ generation: df = 18, *t* = 2.3, *p* < 0.05; 40th generation: df = 18, *t* = 6.0, *p* < 0.0001) (Fig. S1).

### *S*. sp. 1 promote the pathogenicity of PWNs

In this study, we investigated the impact of continuous culture of PWNs on different fungi on their pathogenicity. After 40 generations of continuous culture on a medium inoculated with *S*. sp. 1, PWNs exhibited a wilt rate of 91.67% on pine seedlings. In contrast, after 40 generations of continuous culture on an *O. ips* medium, the PWN showed a wilt rate of 62.5% on pine seedlings (df = 3.2, *t* = 3.5, *p* < 0.05) (Fig. 1C). Moreover, PWNs cultured on *S*. sp. 1 and propagated on pine seedlings for 2912.9 post-generations produced, while the PWN cultured on *O. ips* produced 1599.6 offspring (df = 17.7, *t* = 4.5, *p* < 0.001) (Fig. 1D). These results demonstrate that *S*. sp. 1 can increase both the fertility and pathogenicity of PWNs within host *P. thunbergii*.

### *S*. sp. 1 up-regulated lipid metabolic genes expression of PWN

In this study, we analyzed the impact of *S*. sp. 1 on gene expression of PWN. We observed that CNPWN treatment with *S*. sp. 1 resulted in 170 up-regulated genes (*S*. sp. 1 > *O. ips*) and 212 down-regulated genes (*S*. sp. 1 < *O. ips*); similarly, USPWN treatment with *S*. sp. 1 resulted in 440 up-regulated genes (*S*. sp. 1 > *O. ips*) and 197 down-regulated genes (*S*. sp. 1 < *O. ips*) (Fig. S2). KEGG functional enrichment analysis of these genes revealed that in addition to up-regulating signaling pathways related to reproduction, such as oocyte meiosis and oxytocin signaling pathway, some pathways related to nutrient metabolism were also significantly enriched (e.g., insulin signaling pathway, alcoholism, fatty acid degradation, and glycolysis/gluconeogenesis) (Fig. 1E).

Reproduction and nutrition are closely linked. The nutrient supply plays a vital role in providing energy for reproduction. Organisms undergoing reproduction can mobilize stored energy to improve metabolic efficiency as well. To explore the underlying factors responsible for the increased pathogenicity of PWN, we performed a positive co-expression analysis of these differential genes and found highly similar positive correlations in the expression profiles of genes related to reproduction and lipid metabolism in *S*. sp. 1-treated CNPWN and USPWN (Fig. 1F). Therefore, our findings suggest that *S*. sp. 1 may increase the fecundity of PWN through up-regulation of lipid metabolic genes.

### Identification and expression analysis of lipid metabolism genes

To better understand the impact of *S*. sp. 1 on the fecundity of PWN and identify essential genes or compounds involved, we conducted a genomic search to identify PWN genes associated with lipid metabolism (Fig. 1G). Similar to *C. elegans*, PWN contain different classes of lipids, such as TAG, FAs, and phospholipids, and possess genes involved in the synthesis and degradation of these lipids. Notably, unlike *C. elegans*, PWN have only one delta-9 fatty acid desaturase gene, *fat-5*, whereas *C. elegans* has three, *fat-5*, *fat-6*, and *fat-7* [25]. Furthermore, PWN lack the omega-3 fatty acid desaturase gene, *fat-1* [26]. Similarly, unlike *C. elegans*, which has a single gene for *fat-2* that initiates conversion of monounsaturated fatty acids (MUFAs) to polyunsaturated fatty acids (PUFAs), PWN have three such genes, *fat-2.1*, *fat-2.2* and *fat-2.3* [27]. RT-qPCR analysis revealed that *S*. sp. 1-treated PWN exhibited significantly lower expression of *fat-5*, which was down-regulated by 0.09-fold compared to the *O.* ips-treated PWN (df = 4.0, *t* = -7.3, *p* < 0.01). Additionally, the expression levels of *fat-2.2* and *fat-2.3* were up-regulated by 7.52 and 6.02 times compared to controls, respectively, which was statistically significant (df = 5.0, *t* = 8.0, *p* < 0.0001; df = 4.2, *t* = 7.6, *p* < 0.01) (Fig. 1H). Collectively, these results suggest that FAs, especially MUFAs or their related genes, may be crucial for the reproductive capacity of *S*. sp. 1-treated PWNs.

### *S*. sp. 1-treated PWNs contain a greater abundance of lipid compounds

**Fig. 2:**
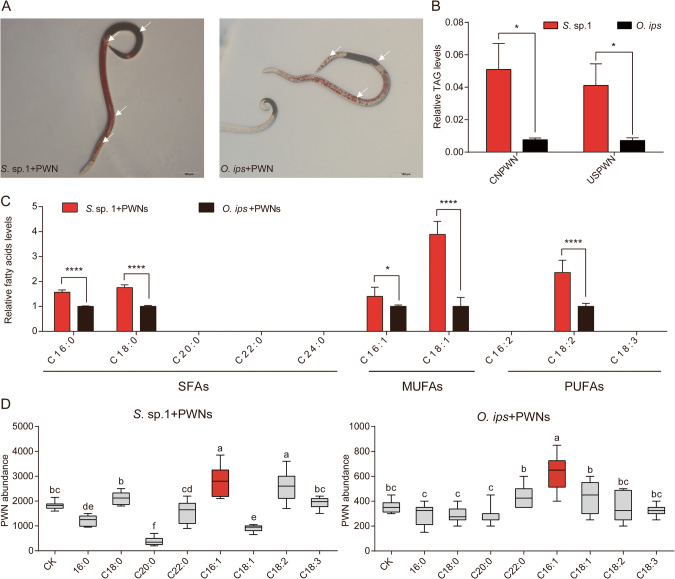
Effect of two blue-stain fungi on lipid metabolism of PWNs. **A** Fat content visualized by oil-Red-O staining. Nematodes were grown and treated as described in Materials and Methods. Staining was performed on female nematodes and photographed using DIC microscope. Red indicates lipid. **B** The relative TAG levels between two blue-stain fungi cultured CNPWN and USPWN. [*n* = 5; *p < 0.05; Student’s *t* test]. **C** The relative fatty acid levels between two blue-stain fungi cultured CNPWN [*n* = 6; *p < 0.05; ****p < 0.0001; Student’s *t* test]. **D** Fecundity of PWNs in the different supplementation of fatty acids (*n* = 12). Labels with different letters are significantly different at *p* < 0.05 (ANOVA, Tukey’s multiple comparison test).

Lipids are mainly stored in LDs in the form of TAGs from nematodes to mammals. This study compared lipid content by staining LDs with oil-red and measuring TAGs. The results of LD staining demonstrated a higher density of LDs in PWNs cultured with *S*. sp. 1 than *O. ips* (Fig. 2A). Correspondingly, PWNs cultured on *S*. sp. 1 exhibited approximately four times higher relative TAG content compared to PWNs cultured on *O. ips* no matter CNPWN and USPWN (df = 5.0, *t* = 2.7, *p* < 0.05; df = 5.2, *t* = 2.5, *p* < 0.05) (Fig. 2B). These findings suggest that PWNs cultured on *S*. sp. 1 may have received more energy for various biological activities as a result of enhanced lipid accumulation.

Furthermore, we determined the FFAs composition of PWNs cultured on two blue-stain fungi by GC/MS, and found that it was mainly composed of SFAs (C16: 0 and stearic acid: C18: 0), MUFAs (C16: 1 and C18: 1), and PUFAs (linoleic acid: C18: 2). The CNPWNs cultured in *S*. sp. 1 had higher levels of FFAs, especially C16: 1 and C18: 1, with fold increases of 1.4 and 3.9, respectively, compared to the control group (df = 10, *t* = 2.5, *p* < 0.05; df = 10, *t* = 6.9, *p* < 0.0001) (Fig. 2C). In addition, the C16: 1 levels is 1.7 fold of the USPWNs cultured in *S*. sp. 1 compared to the control group (df = 7.7, *t* = 3.4, *p* < 0.05) (Fig. S3). Notably, in the previous section, we found that the expression of *fat-5*, which encodes for the synthesis of MUFAs, was downregulated in PWNs cultured in *S*. sp. 1, while the expression of *fat-2.2* and *fat-2.3*, which are involved in the conversion of MUFAs to PUFAs, was upregulated (Fig. 1G). These results suggest that the high levels of MUFAs in *S*. sp. 1-cultured PWNs may not be attributed to biosynthesis but instead to food intake or other routes.

### C16: 1 promote the fecundity of PWNs

In order to investigate the potential role of FAs in enhancing the fertility of PWN, we added various types of FAs to the medium of two fungi. Our research findings demonstrate that the addition of C16: 1 on *S*. sp. 1 increased the PWN number from 1825 to 2779 per Petri dish; Similarly, the addition of C16: 1 on *O. ips* increased the PWN number from 354.2 to 616.7 (PWNs+*S*. sp. 1: *F*_8, 99_ = 14.9, *p* < 0.05; PWNs+*O. ips*: *F*_8, 99_ = 62.7, *p* < 0.05 ANOVA, Tukey’s test) (Fig. 2D). These results indicate that supplementing with C16: 1 improves the fecundity of PWN.

### Gene expression profiles of lipid metabolism in PWNs at different life-stages

**Fig. 3:**
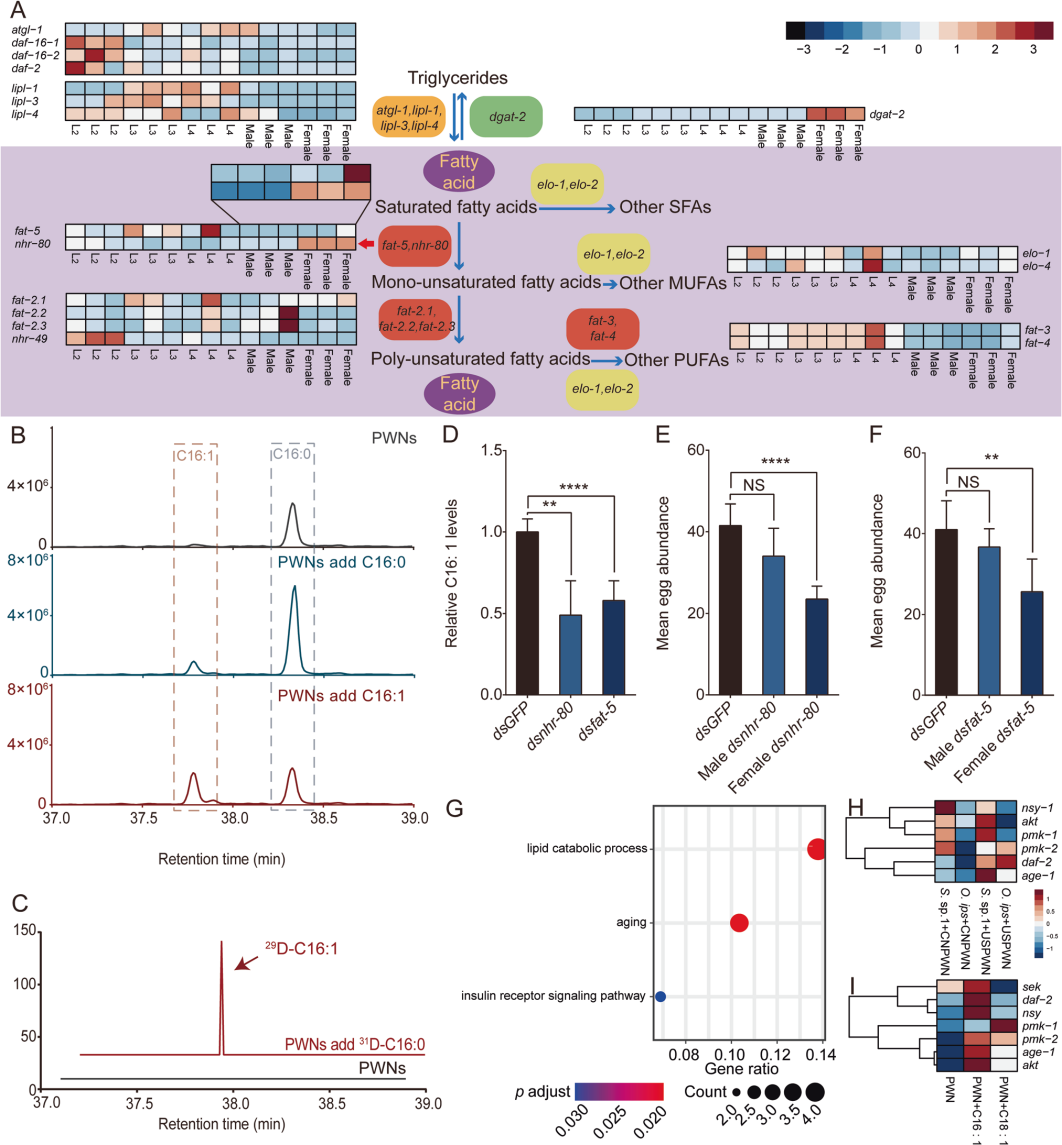
Effect of the C16: 1 produced by female PWNs on fecundity. **A** Expression patterns of lipid metabolism genes in different life-stages of PWN. **B** Biosynthesis and absorbing of C16: 1 by PWNs. Detection of C16: 0 and C16: 1 of PWNs by GC/MS added with C16: 0 or C16: 1. **C** Biosynthesis of C16: 1 by PWNs. Detection of ^29^D-labeled C16: 1 (m/z 298.6) by GC/MS in PWNs fed with ^31^D-labeled C16: 0 (m/z 301.6). **D** Relative C16: 1 levels of PWNs after RNAi by soaking *dsnhr-80*, *dsfat-5*, and *dsGFP* (Control) (*n* = 6). **p < 0.01; ****p < 0.0001; Student’s *t* test. **E** Fecundity was assessed following RNAi of male and female PWNs (6 replications) by soaking with *dsnhr-80* and *dsGFP* (Control) separately. ****p < 0.0001; Student’s *t* test. **F** Fecundity was assessed following RNAi of male and female PWNs (6 replications) by soaking with *dsfat-5* and *dsGFP* (Control) separately. **p < *0*.01; Student’s *t* test. **G** Distribution of GO function groups within up-regulated genes in PWNs treated by C16: 1, *p* adjust< 0.05. **H** Expression patterns of insulin receptor signaling pathway genes treated with *S*. sp. 1 and *O. ips* of PWN based on RT-qPCR. **I** Expression patterns of insulin receptor signaling pathway genes treated with C16: 1 and C18: 1 of PWN based on RT-qPCR.

In addition to dietary acquisition, de novo synthesis is an important way of acquiring FAs. To further investigate the role of C16: 1 in the reproduction of PWN, we compared the expression profiles of lipid metabolism genes in PWN at different life stages. We found that most lipid metabolism genes are highly expressed in the larval stage, but *dgat-2* and the nuclear hormone receptor *nhr-80*, which are associated with TAG and MUFAs content, respectively, are highly expressed in adult stages, particularly in females (Fig. 3A). It has been shown that *fat-5* is a key target of *nhr-80* in *C.elegans*, which is homologous to mammalian *hnf-4*, and can regulate the desaturation of SFAs to MUFAs by modulating the expression of *fat-5* [28, 29]. We found that the promoter region of the *fat-5* gene in PWN contains the putative HNF4 binding domain CAAAGTCCA (Table S2, Fig. S4A). Additionally, we compared the expression of *nhr-80* and *fat-5* between males and females using qRT-PCR and found that the expression level of *nhr-80* in male is 0.68 of that in females (df = 7, *t* = 3.0, *p* < 0.05) and *fat-5* in males is 0.46 of that in females (df = 8, *t* = 7.0, *p* < 0.0001) (Fig. S4B). The C16: 1 content of female and male PWNs was further determined using GC/MS analysis, revealing a higher concentration of C16: 1 in female PWNs (Fig. S4C). These findings indicate that the gene expression involved in the biosynthesis of C16: 1 is more active in female nematodes, suggesting its potential involvement in the reproductive process of PWNs.

### Production and absorption of C16: 1 by PWNs

To confirm the capability of PWN to obtain MUFAs through direct feeding and biosynthesis, the present study supplemented media with C16: 0 and C16: 1 to cultivate PWNs. Specifically, PWNs supplemented with C16: 1 showed a significant increase in their C16: 1 content. Additionally, PWNs fed with C16: 0 were able to produce additional C16: 1 (Fig. 3B).

To provide additional evidence of PWNs’ ability to produce C16: 1, we labeled ^31^D-C16: 0 and assessed whether the label was integrated into C16: 1. The GC/MS analysis revealed the presence of ^29^D-C16:1 in PWNs (Fig. 3C).

### RNAi of *nhr-80* and *fat-5* reduces C16: 1 conversion and decreases PWN fecundity

The ability of PWN to convert C16: 1 by biosynthesis and the high expression of these genes in females were already demonstrated. To further explore how fatty compounds are involved, we performed RNAi of *nhr-80* and *fat-5* on PWNs. Our results showed that both *nhr-80* and *fat-5* knockdowns reduced the expression of *fat-5* and the relative amount of C16: 1 in nematodes (Fig.3D and S5). Notably, the RNAi treatment had sex-specific effects on PWN egg production. For *nhr-80*, the mean egg production of PWNs for both males and females treated with *dsGFP* was 41.5. However, when only males were treated with *dsnhr-80*, the mean egg production reduced to 34 (df = 10, *t* = 2.1, *p* = 0.06), while for females treated with *dsnhr-80*, the egg production further decreased to 23.5 (df = 10, *t* = 7.1, *p* < 0.0001) (Fig. 3E). For *fat-5*, the mean number of eggs laid by *dsGFP*-treated PWNs was 41. However, for males treated with *dsfat-5*, the mean number decreased to 36.7 (df = 10, *t* = 1.3, *p* = 0.238), and for females treated PWNs, it reduced to 25.7 (df = 10, *t* = 3.5, *p* < 0.01) (Fig. 3F). These findings suggest that the *fat-5* gene regulates C16: 1 conversion and the fecundity of PWN females and is regulated by *nhr-80*.

### C16: 1 may improving insulin receptor sensitivity in PWN

In order to gain a deeper understanding of the molecular mechanisms underlying the enhanced fecundity of PWN by C16: 1, we conducted transcriptome sequencing on PWNs treated with both C16: 1 and C18: 1. Our results indicated that PWNs treated with C16: 1 exhibited up-regulation and enrichment of genes associated with lipid catabolism, aging, and the insulin receptor signaling pathway (Fig. 3G). Conversely, the aforementioned pathways were not activated by C18: 1 treatment (Fig. S6). We further validated the up-regulation of genes associated with the insulin receptor signaling pathway using RT-qPCR on PWNs treated with two different fungi for 40 generations, as well as PWNs treated with two different MUFAs. These results showed that both *S*. sp. 1 and C16: 1 were capable of up-regulating genes related to the insulin receptor signaling pathway (Fig. 3H, I).

### MUFAs are more abundant in *S*. sp. 1 compared to *O. ips*

**Fig. 4:**
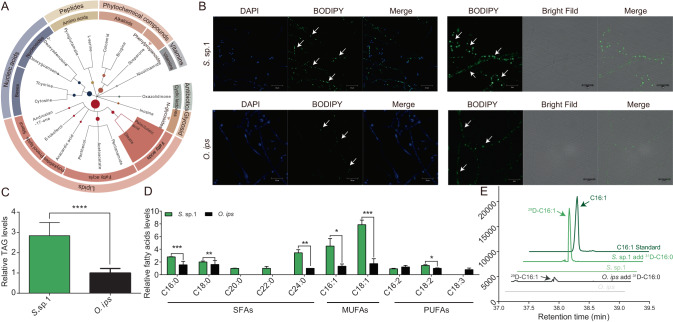
Lipid metabolism of two blue-stain fungi. **A** Analysis of up-regulation metabolite enrichment by *S*. sp. 1. **B** Lipid accumulation of two blue-stain fungi. The mycelia and conidia were stained with BODIPY and DAPI (green: BODIPY, blue: DAPI). **C** The relative TAG levels between two blue-stain fungi. [*n* = 5; ***p < 0.001; Student’s *t* test]. **D** The relative free fatty acid levels between two blue-stain fungi. [*n* = 5; *p < 0.05; **p < 0.01; ***p < 0.001; Student’s *t* test]. **E** Biosynthesis of C16: 1 by *S* .sp. 1 and *O. ips*. Detection of ^29^D-labeled C16: 1 (m/z 298.6) by GC/MS in fungi added with ^31^D-labeled C16: 0 (m/z 301.6). Deuterium labeling slightly increases the retention time of relevant C16: 1.

To investigate the reasons for the higher lipid content in *S*. sp. 1-treated PWNs than in *O. ips*-treated PWNs, and to determine why *S*. sp. 1 is a better symbiotic partner than *O. ips*, we conducted a metabolomic analysis of the metabolites of the two fungi. Using annotation and enrichment analysis of differential metabolites, we observed that lipids, nucleic acids, peptides, phytochemicals, vitamins, and cofactors, which are essential for the growth and development of organisms, were the main categories of metabolites produced by *S*. sp. 1 (up-regulated compared to *O. ips*) (Fig. 4A). Notably, two MUFAs, C16: 1 and C18: 1, were abundant in *S*. sp. 1. Conversely, *O. ips* had a high content of compounds with insecticidal activity, such as atrazine, which can kill nematodes [30], and some alkaloids, including trichothecene, were found to be active ingredients in plant-derived substances with nematicidal activity (Fig. S7) [31]. The interspecific interactions between native symbiotic species and invasive alien species are one of the most critical factors in determining the successful colonization of invasive alien species. Specifically, the fungal species *S*. sp. 1 provides PWNs with relatively high-quality nutrients, while *O. ips* produces compounds that can be harmful to PWNs.

Fungi were subjected to BODIPY staining solution to stain the TAGs in their mycelium and conidia. Bright green fluorescence was observed under the laser confocal scanning microscope, dotting the mycelium and conidia cells. The nuclei were stained bright blue with DAPI. Mycelium was photographed using bright field. A field of view with similar cell numbers was selected for photography, and the green fluorescence of *S*. sp. 1 was densely distributed in the field, indicating its high fat content (Fig. 4B). Measuring the TAG content of the two fungi revealed that *S*. sp. 1 had a TAG content about two times higher than that of *O. ips* (df = 8, *t* = -6.0, *p* < 0.0001) (Fig. 4C).

Furthermore, by using GC/MS to determine the FAs content of the two blue-stain fungi, we identified a range of SFAs with lengths from C16 to C24, including C16: 0, C18: 0, eicosanoic acid (C20: 0), docosanoic acid (C22: 0), and lignic acid (C24: 0); MUFAs were C16: 1 and C18: 1; PUFAs were hexadecanoic acid (C16: 2), C18: 2, and linolenic acid (C18: 3). The relative contents of C16: 0, C18: 0, C16: 1, C18: 1, and C18: 2 in *S*. sp. 1 were 1.81, 1.25, 3.38, 4.52, and 1.45 times higher than those in *O. ips*, respectively (df = 7, *t* = 24.1, *p* < 0.001; df = 7, *t* = 5.6, *p* < 0.01; df = 4.9, *t* = 3.1, *p* < 0.05; df = 7, *t* = 8.1, *p* < 0.001; df = 7, *t* = 2.9, *p* < 0.05;), with C16: 1 and C18: 1 being particularly abundant (Fig. 4D). In order to eliminate any bias introduced by artificial media on fungal fatty acid content, we prepared a pine xylem powder medium to cultivate the blue-stain fungi. The results showed that *S*. sp. 1-cultured in xylem powder medium contained 3.54 times more C16: 1 than *O. ips* (df = 10, *t* = 5.208, *p* < 0.0001) (Fig. S8).

### The native symbiotic fungus *S*. sp. 1 is more capable of produce C16: 1

*Sporothrix*. sp. 1 as a new symbiotic fungi for PWNs, provided more C16: 1 for PWNs, which can significantly promote the fecundity. This suggests that the ability of symbiotic fungi to produce C16: 1 plays a crucial role in the successful invasion of PWNs in China. To confirm that *S*. sp. 1 has a greater capacity to produce C16: 1, we cultured two blue-stain fungi in ME supplemented with ^31^D-C16: 0 and examined if the label was incorporated into C16: 1. The results indicated that ^29^D-C16: 1 was present in both fungi, but was found in greater abundance in *S*. sp. 1 (Fig. 4E).

### C16: 1 promotes mycelial branching and spore production in blue-stain fungi

**Fig. 5:**
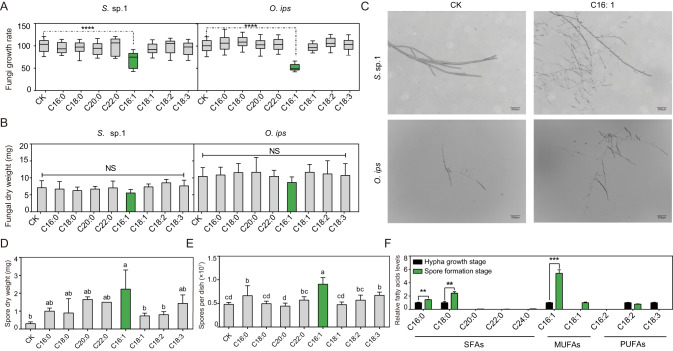
Effect of C16: 1 on two blue-stain fungi. **A** Effects of fatty acids on the growth of *S*. sp. 1 and *O. ips*, CK stands for control. [*n* = 9; ****p < 0.0001; Student’s *t* test]. **B** Effects of fatty acids on the dry weight of *S*. sp. 1 and *O. ips* (*n* = 9), CK stands for control, NS means no significance. **C** Induction of hyphal branching by C16: 1, CK stands for control. **D** Effects of fatty acids on the spore dry weight of *S*. sp. 1, CK stands for control (*n* = 3). Labels with different letters are significantly different at *p* < 0.05 (ANOVA, Tukey’s multiple comparison test). **E** Effects of fatty acids on the sporulation of *S*. sp. 1 (*n* = 15). Labels with different letters are significantly different at *p* < 0.05 (ANOVA, Tukey’s multiple comparison test). **F** The relative fatty acid levels between different life-stages of *S*. sp. 1 (*n* = 4). **, *** stand for significant difference under *p* < 0.01, *p* < 0.001 respectively [Student’s *t* test].

FAs are crucial nutrients for fungal survival. To investigate the effects of various FAs on the growth and development of blue-stain fungi in the PWNs-fungi mutualistic symbiosis system, we used mycelial growth rate and fungal dry weight as criteria to measure the growth and development of fungi. Our results showed that in *S*. sp. 1-treated with C16: 1, the mycelial growth rate was only 68.13% of the control group (df = 94.2, *t* = 10.4, *p* < 0.0001). In *O.* ips-treated with C16: 1, the mycelial growth rate was only 50.47% of the control group (df = 103.2, *t* = 27.6, *p* < 0.0001). While having no significant effect on fungal dry weight (Fig. 5A,B).

Subsequently, we used the paper disc method to investigate the impact of FAs on the mycelial branching of two blue-stain fungi and discovered that C16: 1 promotes fungal mycelial branching (Fig. 5C, S9). Additionally, C16: 1 promoted the production of more spores by the fungi. The dry weight of spores in the C16: 1 treatment was 2.23 mg, compared to 0.3 mg in the control group (*F*_8, 18_ = 3.6, *p* < 0.05). The number of spores in the C16: 1 treatment was 9.06 × 10^6^, while in the control group it was 4.79 × 10^6^ (*F*_8, 126_ = 628.2, *p* < 0.05) (Fig. 5D, E). Fungal life cycle encompasses hypha growth stage and spore formation stage. During hypha growth, fungi absorb more nutrients, while spore formation facilitates fungal reproduction and dissemination. We also examined the FAs composition of *S*. sp. 1 at different life stages. The C16: 1 level during the spore formation stage was 5.4 times higher than during the hypha growth stage (df = 6, *t* = -8.344, *p* < 0.001) (Fig. 5F). The results showed that positive effect of C16: 1 on reproductive capacity is further amplified through a positive feedback loop, with reproductive stages producing and accumulating C16: 1, which, in turn, leads to further enhancement of reproductive capacity.

## Discussion

Metabolites were found to act as “molecular bridges” that facilitate the coordination of symbiotic relationships and the maintenance of homeostasis. In the symbiotic interaction between PWN and fungi, lipids C16: 1 produced by the fungus play a crucial role in enhancing the fecundity and sporulation capacities of both associated partners, respectively, thereby facilitating the establishment of a symbiotic relationship. PWN utilize C16: 1 synthesized by *S*. sp. 1 and PWN themselves to increase fecundity and expand populations. With the host pine trees weakened, there was a lack of sufficient nutrients. The blue-stain fungus might increase its chances of survival by producing spores. The blue-stained xylem with *S*. sp. 1 became the main nutrients of PWN subsequently. Fatty acids have been identified as important signaling molecules in many symbiotic relationships. For plants and symbiotic arbuscular mycorrhizal fungi, FAs are essential energy substrates in the evolution of mutually beneficial symbiotic systems during the process of plant terrestrialization [32]. However, plants must rely on specific membrane transport proteins to effectively convert FAs into acylated glycerol and transport them into the fungal partner to maintain their symbiotic relationship [33]. In contrast, animals can acquire FAs from their symbiotic partners simply by consuming them. The key lipid metabolite C16: 1 plays a crucial role in facilitating the rapid successful colonization of a mutually beneficial symbiotic relationship between the invasive PWN and the native *S*. sp. 1. within the host. It highlights the efficient sharing significance of metabolites and its functions to promote the synchronization of partners in symbiotic relationships.

Understanding the colonization of symbiotic relationships in the introduced ranges is crucial for comprehending colonization and outbreak of invasive species. Our research revealed that when the PWN is introduced, it rapidly utilizes the metabolic resources of the native ophiostomatoid fungi, *S*. sp. 1. This utilization allows the PWN to quickly overcome the bottleneck of a small population by significantly enhancing its reproductive capacity. These metabolites mediated coordination and communication between the symbiotic partners. The ability of symbiotic microorganisms to confer new traits to their partners has also been observed in other invasive species. For example, soil microorganisms in the European cornflowers’s native habitat inhibit its growth and limit its expansion. However, when this cornflowers invades North America and establishes a symbiotic relationship with new soil microorganisms, these microorganisms promote its growth and development [34]. Therefore, studying the key metabolites involved in these symbiotic relationships is a new direction and a new view for elucidating the establishment and spread of invasive species.

In this study, we present evidence that C16: 1 plays a significant role in enhancing the reproduction. Although MUFAs’ positive effects on disease and aging have been widely investigated [35], our findings highlight a novel role for them in improving reproductive capacity. Specifically, we observed that the supplementation of C16: 1 promotes sporulation by *S*. sp. 1 and reproduction by PWNs. For *S*. sp. 1, C16: 1 producing and accumulating during the spore formation phase compared to the hypha growth stage. For PWN, the up-regulation of *nhr-80* and *fat-5* in female promotes the biosynthesis of C16: 1. Furthermore, our findings suggest that C16: 1 may promotes nematode fecundity by enhancing PWNs’ regulation of the insulin signaling pathway. C16: 1 also has been reported to improve insulin sensitivity in humans, and the insulin signaling pathway has been shown to regulate organisms’ reproductive capacity of mating, egg production, and ovarian development [36, 37]. The MUFAs' role of getting offspring might be conserved in organisms and merits further investigation.

The results of our study provide further support for the idea that "you are what you eat." It’s indicated that the nutritional quality of the food source might have a direct impact on the reproductive outcomes. The relationship between diet and fertility in a variety of organisms are also showed in previous studies. For instance, in zebrafish, docosahexaenoic acid (DHA) enhances fertility, while arachidonic acid (ARA) broadly promotes fertility in birds [38]. In addition, diet can also influence biological processes such as metabolism, immunity and development of organisms through gene transfer, signal regulation, and reshaping of the gut microbiota [39–41]. The positive effects of MUFAs are particularly noteworthy, given their dietary availability of using diet as a tool to improve reproductive outcomes in both humans and other organisms. Indeed, proteomic and carbohydrate effects on the fecundity of PWN may also have a dramatic effect and deserve further investigation.

### Supplementary information

SUPPLEMENTAL MATERIAL

## Data Availability

The datasets presented in this study can be found in online repositories. The names of the repository/repositories and accession number(s) can be found below: National Center for Biotechnology Information (NCBI) BioProject database under Accession Number PRJNA941926. All other study data are included in the article and/or supporting information files.
