# Supplementary material for: Palmitoleic acid as a coordinating molecule between the invasive pinewood nematode and its newly associated fungi
Source: ISME J. 2023 Aug 21;17(11):1862–71. doi: 10.1038/s41396-023-01489-8 (PMC10579226; doi:10.1038/s41396-023-01489-8)
Supplement: Supplementary file 1 — SUPPLEMENTAL MATERIAL [file 41396_2023_1489_MOESM1_ESM.pdf]

Palmitoleic acid as a coordinating molecule between the invasive pinewood nematode and its newly associated fungi

Jing Ning, Xiaoting Gu, Jiao Zhou, Hongxia Zhang, Jianghua Sun, Lilin Zhao

Supplementary Information

Contents

Supplementary Methods 2
- Supplementation of Fatty acids (FAs)..2
- Xylem powder medium..2
- RNA-seq Analyses 2
- Quantitative real-time PCR analysis..3
- Mass spectrometry 3
- Effect of FAs on the hypha growth 4
- Paper disk diffusion assay..4
- Effect of FAs on the sporulation 4
- Statistical analysis..5
- References..5

Table S1 Primers used in this study 6
Table S2 The promoter region of the *fat-5* gene in PWN 8
Figure S1 The USPWN abundance continuously cultured on two blue-stain fungi for the 0, 5th, 20th and 40th generations 9
Figure S2 The number of up- and down- regulated genes of CNPWN and USPWN cultured by two blue-stain fungi 10
Figure S3 The relative fatty acid levels between two blue-stain fungi cultured USPWNs 11
Figure S4 Differences in C16: 1 and related genes in males and females 12
Figure S5 Relative expression of *fat-5* after RNAi by soaking *dsfat-5*, *dsnhr-80*, and *dsGFP* (Control) in males and females..13
Figure S6 Distribution of GO function groups within up-regulated genes in PWNs treated by C18: 1 14
Figure S7 Enrichment analysis of up-regulated metabolites by *O. ips*..15
Figure S8 Relative C16: 1 levels of fungi cultured on xylem powder medium 16
Figure S9 Induction of hyphal branching by different kinds of fatty acids. 17

Supplementary Methods

Supplementation of Fatty acids (FAs)

The protocol for supplementing FAs was adapted from a previous study with modifications. Tergitol detergent (type NP-40, Sigma-Aldrich) was added to the MEA medium at a final concentration of 0.1% for both non-supplemented and supplemented plates prior to autoclaving, facilitating FA dissolution. During the initial experimental optimization, FAs were used at concentrations ranging from 0.1 mM to 5 mM, and a consistent concentration of 1 mM was employed for all subsequent supplementation experiments. GC/MS analysis confirmed the successful incorporation of supplemented FAs into the blue-stain fungi and PWNs.

For PWNs, the supplementation assay with C16: 0 and ^{31}D-labeled C16: 0 was conducted on 2% PDA medium (20 g PDA, 15 g agar, and 1 L deionized water) using *Botrytis cinerea* as the plate culture. C16: 0 and ^{31}D-labeled C16: 0 were dissolved in ethanol to a concentration of 1 mM and evenly spread in 50 μL aliquots on the fungal mat. Approximately 1000 surface-sterilized PWNs were applied to the plates with ^{31}D-labeled C16: 0, and an additional 1000 PWNs soaked in dsRNA were applied to the C16: 0 plates for a duration of one week. Nematodes were counted using the Baermann funnel method to determine changes in fecundity and collect samples for GC/MS analysis. For blue-stain fungi, the supplementation assay with and ^{31}D-labeled C16: 0 was conducted on 2% MEA medium.

Xylem powder medium

The xylem powder medium was prepared by mixing 20 g of dry *P. thunbergii* xylem powder (The xylem was dried, ground and sieved at 80 mesh) and 10 g of agar followed by 300 mL of distilled water, which was autoclaved (30 min, 120 °C, 0.14 Mpa) then poured into Petri dishes (1).

RNA-seq Analyses

PWNs were pooled in a 1.5 mL tube and either used immediately for RNA extraction or stored at −80 °C until further use. PWNs were disrupted using a Cell crusher in 500 μL of TRIzol (Invitrogen, Carlsbad CA, USA), and total RNA was extracted following the Trizol protocol (Invitrogen). RNA integrity was assessed using a NanoDrop ND-1000 spectrophotometer (NanoDrop Technologies, Inc., Wilmington, DE 19810, USA), and the A260/A280 ratio of the RNA was maintained between 1.8 and 2.0.

MRNA purification was performed from each sample using the Dynabeads mRNA purification kit (RiboPureTM Kit Family Ambion, Life Technology, Waltham, MA, USA). RNA-seq libraries were prepared using the RNA-Seq Library Preparation Kit for Whole Transcriptome Discovery and sequenced on an HiSeq 2000 (Illumina) platform at the Beijing Institute of Genomics (Chinese Academy of Sciences) (2).

The original sequences from each cDNA library underwent preprocessing to eliminate low-quality sequences, adaptors, and reads contaminated with microbes. The clean sequences were *de novo* assembled into contigs using the Trinity program (version 2013) with default parameters. Following assembly and removal of redundant sequences, the clean reads were clustered using the Cd-hit clustering software. For functional annotations, all unigenes were searched against the NCBI non-redundant

(NR) sequence database (http://www.ncbi.nlm.nih.gov/) and Swiss-Prot database using BLASTX (E-value < 10-5). Additionally, gene ontology (GO) enrichment analysis was performed on all unigenes using BLAST2GO against the GO database to confirm enriched GO terms. Lastly, enriched pathways and metabolic networks were identified using KEGG (Kyoto Encyclopedia of Genes and Genomes database) analysis, which was performed on the KEGG website. Fragments per kilobase of exon per million fragments mapped (FPKM) values were calculated using the R package RSEM (3). Differentially expressed genes (DEGs) were filtered using the R packages DEGseq and DEseq2 with a cut-off p-value < 0.05 and a fold change greater than 2 or less than 0.5 (4, 5). Graphlan was used to generate a circular representation of the enrichment tree for the specified genes (6). A gene co-expression network was constructed for highly expressed genes in the two co-expression groups treated with *Sporothrix* sp. 1 and *Ophiostoma ips* in CNPWN and USPWN. This network was based on pairwise gene expression correlations determined by calculating the Pearson correlation coefficient (r) using R software (7). The network was constructed by connecting genes with an r-value ≥ 0.80 to minimize false connections resulting from weak correlations between genes. The resulting co-expression networks were visualized using CYTOSCAPE software (8).

Quantitative real-time PCR analysis

To confirm the expression profile obtained from RNA-seq results, we selected some of the major lipid-related genes and insulin signal-related genes listed in Fig. 1G, and designed specific primers (Table S1) for performing a quantitative real-time PCR analysis. A total of 1 μg RNA was used for cDNA synthesis using the FastQuant RT Kit (Tiangen, China). Each reaction was repeated five times as replicates. The quantitative real-time PCR was performed on an MX3000P system using SYBR green PCR Master Mix. The thermal cycling conditions were as follows: 95 ºC for 10 s, 55 ºC for 20 s, and 72 ºC for 30 s (40 cycles). Relative expression levels were normalized using the internal control gene encoding β-actin and calculated using the comparative $2^{-\Delta\Delta CT}$ method (9).

Mass spectrometry

The GC was equipped with an HP-5MS column [60 mm × 0.25 mm (i.d.); film thickness 0.25 μm] (J&W Scientific). One-microliter samples were injected at a temperature of 280 °C; the GC/MS transfer line temperature was 280 °C, ion source set to 230 °C, and quadrupole set to 150 °C. All compounds were analyzed with 70 eV nominal electron energy and a scan range of 35–400 atomic mass units, with a solvent delay of 3 min. After injection, the column temperature was held at 50 °C for 0.5 min and then was increased to 200 °C at 5 °C/min, followed by an increase to 240 °C at 2 °C/min and then increased to 250 °C at 5 °C/min and held for 10 min. Subsequently, the temperature was increased further to 280 °C at 3 °C/min and held for 3 min. Compounds were identified by comparing their retention time with those of authentic reference compounds and comparing the spectra with that of mass spectral library NIST02 (Rev. D.04.00; Agilent Technologies).

Metabolomic analysis

25 mg of tissues were homogenized with 800 μL of precooled extraction reagent (methanol: acetonitrile: water, 2:2:1, v/v/v) containing internal standards mix 1 (IS1)

and internal standards mix 2 (IS2) for quality control. After 5 min of homogenization using TissueLyser (JXFSTPRP, China), samples were sonicated for 10 min, incubated at -20 °C for 1 hour, and then centrifuged at 2,5000 rpm for 15 min at 4 °C. The supernatant was vacuum freeze dried, and metabolites were resuspended in 200 µL of 10% methanol, followed by 10 min of sonication at 4 °C. After centrifugation at 2,5000 rpm for 15 min, the supernatants were transferred to autosampler vials for LC-MS analysis. A quality control (QC) sample was created by pooling equal volumes of each sample to assess LC-MS reproducibility.

Samples were analyzed using a Waters 2D UPLC (Waters, USA) coupled to a Q-Exactive mass spectrometer (Thermo Fisher Scientific, USA) with heated electrospray ionization (HESI) source, controlled by Xcalibur 2.3 software (Thermo Fisher Scientific, Waltham, MA, USA). Chromatographic separation used a Waters ACQUITY UPLC BEH C18 column (1.7 µm, 2.1 mm × 100 mm, Waters, USA) at 45 °C. Mobile phase was 0.1% formic acid (A) and acetonitrile (B) for positive mode, and 10 mM ammonium formate (A) and acetonitrile (B) for negative mode. Gradient conditions: 0-1 min, 2% B; 1-9 min, 2%-98% B; 9-12 min, 98% B; 12-12.1 min, 98% B to 2% B; 12.1-15 min, 2% B. Flow rate: 0.35 mL/min, injection volume: 5 µL.

Mass spectrometric settings for positive/negative ionization: spray voltage, 3.8/−3.2 kV; sheath gas flow rate, 40 arb; aux gas flow rate, 10 arb; aux gas heater temp., 350 °C; capillary temp., 320 °C. Full scan range: 70–1050 m/z, resolution 70,000, AGC target for MS set to 3e6, max ion injection time 100 ms. Top 3 precursors selected for MSMS with max ion injection time 50 ms, resolution 1,7500, AGC 1e5. Stepped collision energy: 20, 40, and 60 eV.

Effect of FAs on the hypha growth

We selected 1 mM FAs to assess the effect of different kind of FAs on the growth performance of two blue-stain fungi. Fungal linear growth along the plastic division was measured from the point of inoculation to the leading edge of the hyphae every day, and all treatments were replicated 9 times. At 13 d post-inoculation, the mycelial mat was gently scraped from the agar using disposable blades, and its fresh weight was determined. The mycelia were then allowed to dry at 50 °C until the weight was constant.

Paper disk diffusion assay

Approximately 1000 spores of *S*. sp1 were inoculated on 1% MEA plates and *O. ips* on 2% MEA plates. The plates were incubated at 25 ℃ in the dark. FAs were dissolved in chloroform for 5ug/ml. Two paper disks (3.5 mm in diameter) loaded with FA solutions and chloroform (control) were placed on the plates at 6 h after inoculation. Hyphal branching were observed under a stereoscopic microscope at 24 h after treatment, respectively. All images were taken at 5× magnification.

Effect of FAs on the sporulation

To assess fungal sporulation, 14 day-old the fungal cultures were washed with 10 mL of distilled water and the spore suspension was added to a 50 mL centrifugal tube and vortexed. The number of spores was counted with a haemocytometer. Each sample was counted three times, and each treatment was repeated 5 times. Furthermore, 3 samples concentrated up to dryness in vacuum centrifugal concentrator and count its

dry weight.

Statistical analysis

Oneway ANOVA with Tukey's multiple comparison test was used for analysis of population number, spore number and dry weight treated by FAs. Independent sample *t* test was used for all other assays. GraphPad Prism 5 (GraphPad Software Inc., San Diego, CA, USA) and IBM SPSS 18.0 software (SPSS, Inc., Chicago, IL, USA) were used for statistical analyses. A value of $p < 0.05$ was considered statistically different.

References

1. Zhou FY, Xu LT, Wang SS, Wang B, Lou QZ, Lu M, et al. Bacterial volatile ammonia regulates the consumption sequence of D-pinitol and D-glucose in a fungus associated with an invasive bark beetle. ISME J. 2017;11:2809-20.
2. Hou Y, Wang XL, Saha TT, Roy S, Zhao B, Raikhel AS, et al. Temporal coordination of carbohydrate metabolism during mosquito reproduction. Plos Genet. 2015;11: e1005309.
3. Li B, Dewey CN. RSEM: accurate transcript quantification from RNA-Seq data with or without a reference genome. Bmc Bioinformatics. 2011;12:1-16.
4. Wang LK, Feng ZX, Wang X, Wang XW, Zhang XG. DEGseq: an R package for identifying differentially expressed genes from RNA-seq data. Bioinformatics. 2010;26:136-8.
5. Love MI, Huber W, Anders S. Moderated estimation of fold change and dispersion for RNA-seq data with DESeq2. Genome Biol. 2014;15:1-21.
6. Asnicar F, Weingart G, Tickle TL, Huttenhower C, Segata N. Compact graphical representation of phylogenetic data and metadata with GraPhlAn. PeerJ. 2015;3:e1029.
7. Usadel B, Obayashi T, Mutwil M, Giorgi FM, Bassel GW, Tanimoto M, et al. Co-expression tools for plant biology: opportunities for hypothesis generation and caveats. Plant Cell Environ. 2009;32:1633-51.
8. Shannon P, Markiel A, Ozier O, Baliga NS, Wang JT, Ramage D, et al. Cytoscape: a software environment for integrated models of biomolecular interaction networks. Genome Res. 2003;13:2498-504.
9. Liu H, Wu F, Wu X, Ye J. Differential effects of rapamycin on *Bursaphelenchus xylophilus* with different virulence and differential expression of autophagy genes under stresses in nematodes. Acta Biochim Biophys Sin (Shanghai). 2019;51:254-62.

Table S1 Primers used in this study

Primer name	Primer sequences	Application
atgl-1qF	AGACGCTCATATCCAATGC	lipid metabolism qRT-PCR
atgl-1qR	CACCATCCACATACTCAACT	lipid metabolism qRT-PCR
daf-16-1qF	GAATGGATGGTTCAGAATGTC	lipid metabolism qRT-PCR
daf-16-1qR	TGATAACCCACCAAGATGAC	lipid metabolism qRT-PCR
daf-16-2qF	TACGCCGACTTGATAACTC	lipid metabolism qRT-PCR
daf-16-2qR	CCGACTGTGTAATGATAGGT	lipid metabolism qRT-PCR
daf-2-qF	GCTCATCATCGCTATCCAA	lipid metabolism qRT-PCR
daf-2-qR	GGTAACATCTCCTCGCATT	lipid metabolism qRT-PCR
lipl-1qF	TCTCTTCCGATGTCACCTA	lipid metabolism qRT-PCR
lipl-1qR	AATTATTGCTGCTGGCTTC	lipid metabolism qRT-PCR
lipl-3qF	TCTCCTCGCTTATGATTATGG	lipid metabolism qRT-PCR
lipl-3qR	AAGTCGTTGTGGTTGAAGT	lipid metabolism qRT-PCR
lipl-4qF	CTATACGAGGCTCATCAAGA	lipid metabolism qRT-PCR
lipl-4qR	AATATCCACGACTTCACACT	lipid metabolism qRT-PCR
dgat-2qF	TTGCGATGTATTGCGTATG	lipid metabolism qRT-PCR
dgat-2qR	TCCTTGAATCCAGTTCCATT	lipid metabolism qRT-PCR
fat-5qF	AATGATCCAGCTTCAGAAGT	lipid metabolism qRT-PCR
fat-5qR	AACACCATAGTCTGCCATT	lipid metabolism qRT-PCR
nhr80qF	CCTGCCTATCACTTGTTCA	lipid metabolism qRT-PCR
nhr80qR	CCATCGTCAGATTCATTAGTC	lipid metabolism qRT-PCR
fat-2.1qF	TGTTCTTGCTGGCTTGTAT	lipid metabolism qRT-PCR
fat-2.1qR	TGATGATGCTGTCTGTGAG	lipid metabolism qRT-PCR
fat2.2-qF	GTTCTCGTTGTTTGTTGTTG	lipid metabolism qRT-PCR
fat2.2-qR	GATGACTGTGGTCCTTCTC	lipid metabolism qRT-PCR
fat2.3-qF	CCTTCTCCAGGCTCTTCA	lipid metabolism qRT-PCR
fat2.3-qR	TCCACTCCGAATCCTCATA	lipid metabolism qRT-PCR
NHR-49qF	GATTCTTCCGTCGAACCA	lipid metabolism qRT-PCR
NHR-49qR	ATTGAATGGCTTCCCGTTT	lipid metabolism qRT-PCR
fat-3-qF	TGTCACCATCAGCCATTC	lipid metabolism qRT-PCR
fat-3-qR	TGAACTTGAGGAAGAACTGT	lipid metabolism qRT-PCR
fat-4-qF	GGCAATCTTCTCCAAGGTT	lipid metabolism qRT-PCR
fat-4-qR	TCCAGTAGATGTGTTGATAGG	lipid metabolism qRT-PCR
elo-1qF	TACAGTTATCCATCGCCTAC	lipid metabolism qRT-PCR
elo-1qR	TAGCCATTGATTCCAGAAGT	lipid metabolism qRT-PCR
elo-2qF	GACAACTATCTGATGGACCTT	lipid metabolism qRT-PCR
elo-2qR	TCCTGAGCACCAAGAACA	lipid metabolism qRT-PCR
acl-1qF	TAGAATACACCGTGGAGAAC	lipid metabolism qRT-PCR
acl-1qR	CGAATATGGCGTTACACTTC	lipid metabolism qRT-PCR
acl-4qF	CGAAGGCACTTGTATCAAC	lipid metabolism qRT-PCR
acl-4qR	CGACATTGGCGGAAGATA	lipid metabolism qRT-PCR
acdh-1qF	CTACTTGACCAGACTCCATAA	lipid metabolism qRT-PCR
acdh-1qR	GCCATAACCAGGAACAACT	lipid metabolism qRT-PCR

acdh-2qF	TTCTTCTCTGTTATCCTCACC	lipid metabolism qRT-PCR
acdh-2qR	GCAGCCAATCCAATCCTT	lipid metabolism qRT-PCR
ech-6qF	CGAGGACTTATGAACGAGAT	lipid metabolism qRT-PCR
ech-6qR	ATGATTGGCTTGCGAAGT	lipid metabolism qRT-PCR
hacd-1qF	TGATTGAAGCCATCGTTGA	lipid metabolism qRT-PCR
hacd-1qR	AGGTCTTGTCGTGTCTCA	lipid metabolism qRT-PCR
acox-1qF	AGTCTTATGGCTGATCTACAC	lipid metabolism qRT-PCR
acox-1qR	ATCGTTGGCTCTGATTCG	lipid metabolism qRT-PCR
acox-3qF	TGAGGAAGTCGCAAGGAA	lipid metabolism qRT-PCR
acox-3qR	TTGGAATCGGCTGATGTAG	lipid metabolism qRT-PCR
maoc-1qF	ACATGCCGCTTATTACACT	lipid metabolism qRT-PCR
maoc-1qR	AGCCTTCATTCCACCTAAC	lipid metabolism qRT-PCR
dhs-28qF	AATGGCTCTCGTTGGAATG	lipid metabolism qRT-PCR
dhs-28qR	TCTGTAATGCTTGACCTGAC	lipid metabolism qRT-PCR
daf-22.1qF	CGGAGTTCAAGTCGGATG	lipid metabolism qRT-PCR
daf-22.1qR	GAGTCGGTGCCAATGTAG	lipid metabolism qRT-PCR
daf-22.2qF	TCAATCCAACGACTTCCAT	lipid metabolism qRT-PCR
daf-22.2qR	GAGCACCTCATCCAATGT	lipid metabolism qRT-PCR
daf-22.3qF	TCGCCTTCTCGTCTACAA	lipid metabolism qRT-PCR
daf-22.3qR	CGATGGTGCTGAGTTGAA	lipid metabolism qRT-PCR
EF1-DF	GTTTCTGTCGCATTGAACCTCC	lipid metabolism qRT-PCR
EF1-DR	CGATGGTCAAGAAATAACGGGTAA	lipid metabolism qRT-PCR
NHR80T7F	taatacgactcactatagggCCTGCCTATCACTTGTTCA	RNAi
NHR80T7R	taatacgactcactatagggCCATCGTCAGATTCATTAGTC	RNAi
FAT5T7F	taatacgactcactatagggAATGGCAGACTATGGTGTT	RNAi
FAT5T7R	taatacgactcactatagggAGATTGGATTAGCGTAGAGG	RNAi
GFP-FT7	taatacgactcactatagggGGCAAGCTGACCCTGAAGTT	RNAi
GFP-RT7	taatacgactcactatagggGTCCATGCCGAGAGTGATCC	RNAi
daf-2-qF	GCTCATCATCGCTATCCAA	insulin receptor signaling pathway qRT-PCR
daf-2-qR	GGTAACATCTCCTCGCATT	insulin receptor signaling pathway qRT-PCR
age1-qF	CCTTCTCCACTGAATCCAT	insulin receptor signaling pathway qRT-PCR
age1-qR	ACGCAAGTCATCTCCATT	insulin receptor signaling pathway qRT-PCR
akt-qF	AAGTGCTGGACGATAATGAT	insulin receptor signaling pathway qRT-PCR
akt-qR	TGACAAGTAGATTGCTGAGT	insulin receptor signaling pathway qRT-PCR
sek1-qF	CGCTGTATCGTTGGTAGAT	insulin receptor signaling pathway qRT-PCR
sek1-qR	CTGAACTCCGTATGCTTGT	insulin receptor signaling pathway qRT-PCR
nsy1-qF	CCGATGTCCTCAATCTGG	insulin receptor signaling pathway qRT-PCR
nsy1-qR	ACTGTGTCCTTGTACTTCTC	insulin receptor signaling pathway qRT-PCR
pmk1-qF	CCTCTCCAGAATCTCATCAG	insulin receptor signaling pathway qRT-PCR
pmk1-qR	CTACAGGCTCATCGTTGG	insulin receptor signaling pathway qRT-PCR
pmk2-qF	CCTATACACCTGACACTGAC	insulin receptor signaling pathway qRT-PCR
pmk2-qR	ATGGATTACATCTGCGGAAT	insulin receptor signaling pathway qRT-PCR

Table S2 The promoter region of the *fat-5* gene in PWN

>Bu_scaffold37_cov107_len2094627:629204-631304
ATTTCATCGAGATCAGCCGCCAAGTACTGATTTTGAATAAGGTCATCGACCCGAGTG
TTGTGATCGATGGTCGTTACCTCGAGGCTCGAATCCATGGCCATCTCTATTCGTATAC
AATGATCCTTATTTGTATACAACAATGACTCTCGGATTATTCTGAGTGCCCCTTTTATA
GCCTCTTCCCCTTACTGTAGAAGTGTTCTGAAAAGGGGCGGAGCCAACGGTCATCCC
ATTCGGAAGGGAATTGGTTAAAATAAACATTTTGACTAAATTGGGCGACCCTGAAAT
CAAGGCATCTTGCTATTTTGGCCCCGTATCAGAGAACGAGTTCATCTTTTTCCGGACC
TTGTCCGTGATCCATAATGGGAAGGAGATGGGACCACGGAATTTGCAACCAAATAAC
AATCACTTACGCCTTCTTATGGCTTGAAGATGTGAATTCTTGGAAACAAAAATAATGC
ATGCACTAAGAATGCTATTTCGTAACTCCTGGATTACTGTACTTATTTTTAGAACATTA
TTATATAAGCTAAGACTAAGAAAGTAAGAAAGAGTTGATGTCAACAACGTGATAGTT
GTCTCATGATCTGTAAAATAATTTGCAGGGTGTCTTTGGTCTTTGTTGTTTATAATTTG
GTTGAAAGTGCATTCCGATCTTTTGTGGTACCACTTAAAGTAGACAAAATTAATGTAT
TTATTTGCCTACAGTAACCTCTGATGTCACCAATTGTCTTCCAGATTTGTAGATAATTT
ATTCCAAGGGACTACGAGATAATATTTTGTAGAAAGTACCCAAACTTCTTAGACTCCC
CCGATTCTATCCGCAAACTTCTGAGAATCCAAATTGTGTTCTTATACTATCTATCCATT
TTTGTAAGCAATTACTGTTTGCTGCATGACGTCCATGCATTATGCCCATGAAAACTTT
TAAAATGGCTTCAATTGTAATACAATTAAAATACAGGGCTATTATTCCTCTAAATGATG
ACCAAAATAATGTTGCCGAGTACTTTTATTAAGTCTAAATCCTTCGTAAGCCGGTCTT
AAATGAAGAACGGGGGTTTGATCGCTGATGAGACCACCAGGCAGTCCAATCACACT
AGACTAGAGTGGGGAGAGACGCCGTCAGAACGGGTGGTCAGGTCACGGACTCCGC
CCCCAAGTGGCACCATTCGTTTATCTCCAAAGTCCACGAAGATACACTACACCAGAT
TCATTGTCCATTCCTCGAATCAGCTGTAGAAAGTGCAGAGAATAGGATGATAACAGA
CCGGGTAACTGGATGCAACGTGATCCGACGTCATCATTTACAATGTTTTGCTCACTAT
AGTTAATAGTGATCGGTACTTTGATCCTATTACTCCACTCATCGATCAATCGGATCCAT
TGCAACATTTTGTGCATTGTACCGGCAGCTCTCTTCAAATTCGTTTTATTTAAACGTT
CCTTGATTCTTGTCTGATTTCAAAAAATAATAAATAAAAATGATTCATTTTTTAAAAAT
TACAGAAAACTTTTTATTTTATTGAATGTCAATACGAATTACTTGCACTTTATATTGGG
GAATGGGAGGCGGCAGCAGATAAACGACCGGTCGGATGGACACAAAAATGGGAGT
AGGCGGCGGCCGAGAAGGGGGGAGTTCGGGTATAACATCAGATCTGGAAGGTGGA
GAGCTGCGGTCTGAAAAGAGACAGGGTTCTTACATCGGTATCGGAAATGAACAAAA
CTATCGTGAACAAAGGAATGACCAATGTCTCAAAAGTTAATGAATCATGTAACAATA
GTCTACATACAAACTTCAAAATCATTATTTTTATAAAAATAGATGACTATACCTTTAGC
GTAATATCTCGGTTTAATAAAAGTATCTTTGCGTAGTCTTGGGCGGACTTTGGATCCTT
GGCCAAGTGGATGGGCAGAAGAAGATCGTCGCCGCAACTTCACCTCCTTCCGAACC
CCCTCAGACATCATGAACAAGGTGAATAAAGGAGGGAATGAAAAGGGAATTAATCA
AATCAATGATAACGAGGAATTAAGTCAATCAGCCGAGCCGGAGCTCCCCCGTTTTTT
GAAGGGATTGAGGAAGTTGATGGCCTCG

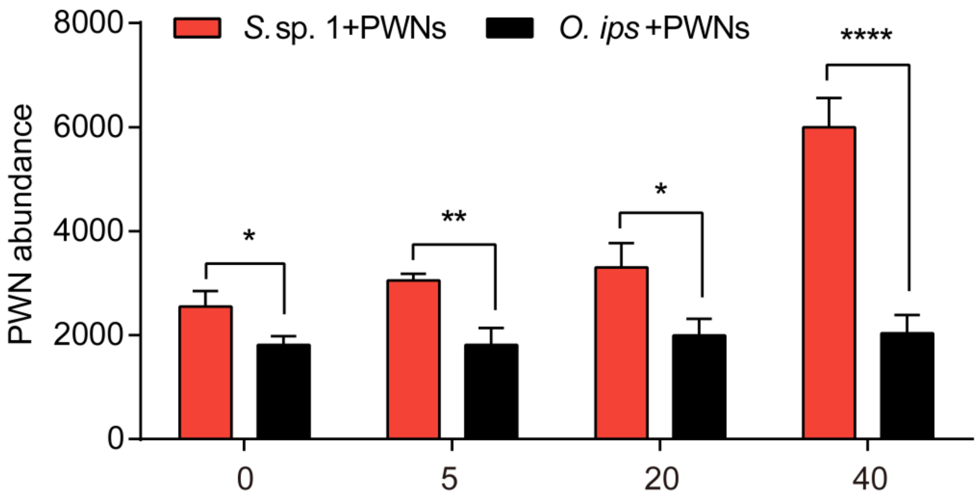

Figure S1 The USPWN abundance continuously cultured on two blue-stain fungi for the 0, 5th, 20th and 40th generations, and 10 nematodes were inoculated into each dish, with 10 replicates for each treatment. *, **, **** stand for significant difference under $p < 0.05$, $p < 0.01$, and $p < 0.0001$ respectively [Student's t test].

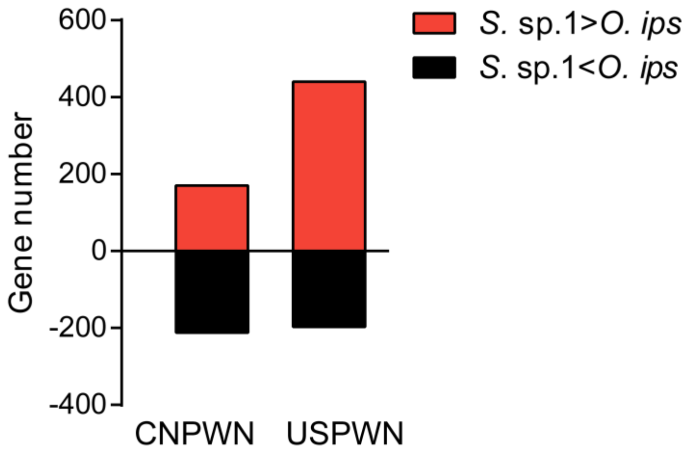

Figure S2 The number of up- and down- regulated genes of CNPWN and USPWN cultured by two blue-stain fungi

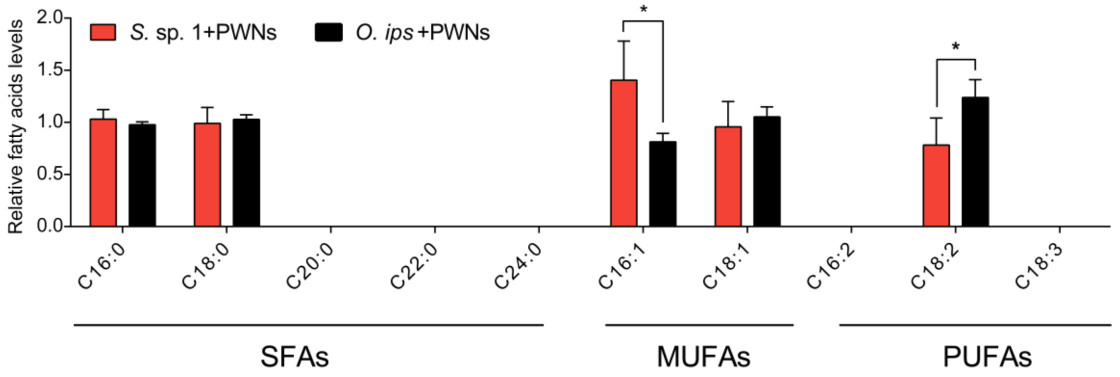

Figure S3 The relative fatty acid levels between two blue-stain fungi cultured USPWNs [*n*=6; *, $p < 0.05$; Student's *t* test].

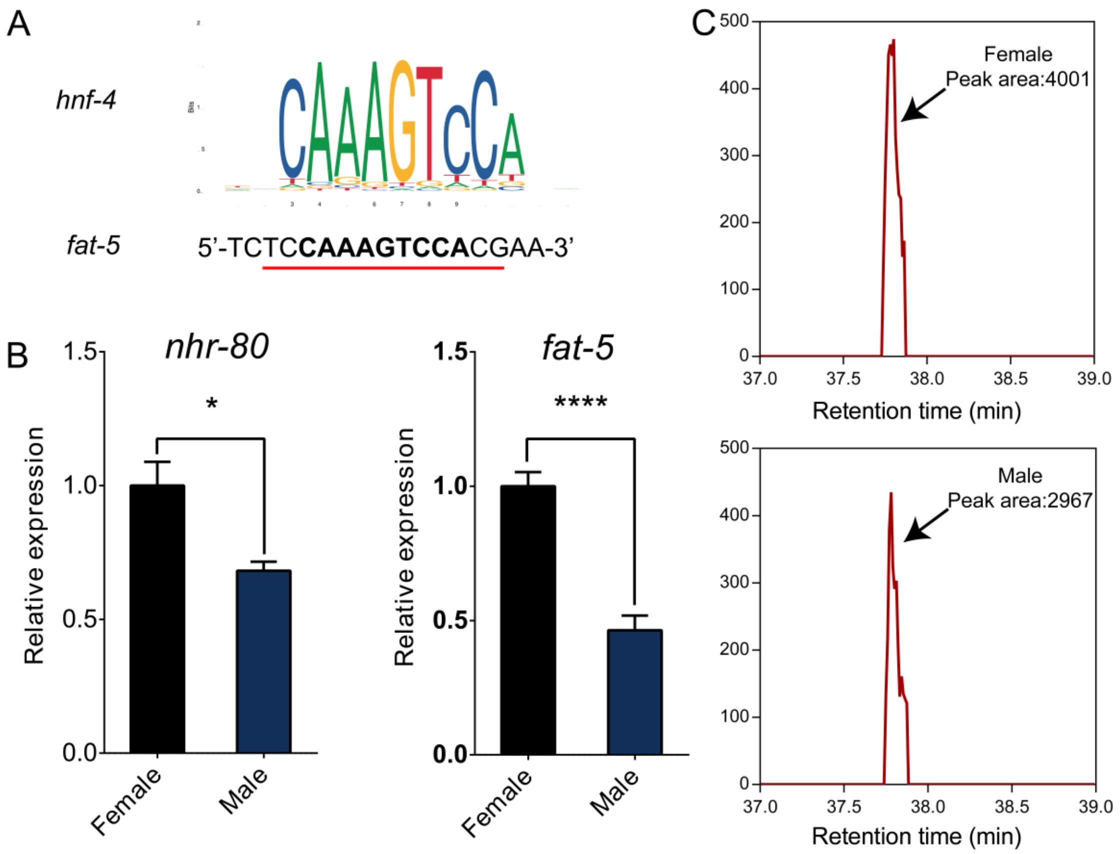

Figure S4 Differences in C16: 1 and related genes in males and females (A) Promoter region of the *fat-5* gene in PWN contains the putative HNF4 binding domain CAAAGTCCA (B) Relative *nhr-80* and *fat-5* levels of PWNs between males and females (n=6). *, p < 0.05; ****, p < 0.0001; Student's t test. (C) Male and female PWN C16: 1 content.

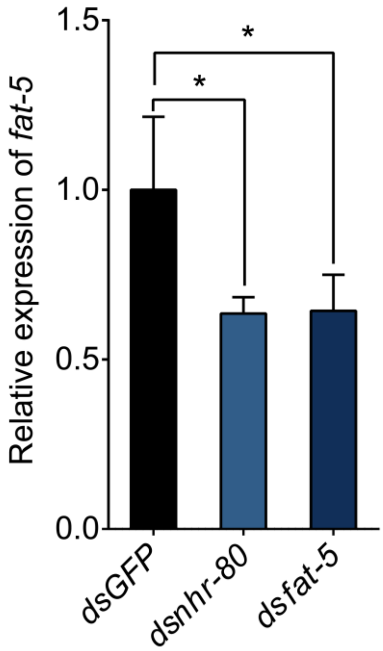

Figure S5 Relative expression of *fat-5* after RNAi by soaking *dsfat-5, dsnhr-80*, and *dsGFP* (Control) in males and females (*n*=6). *, $p < 0.05$; Student's *t* test.

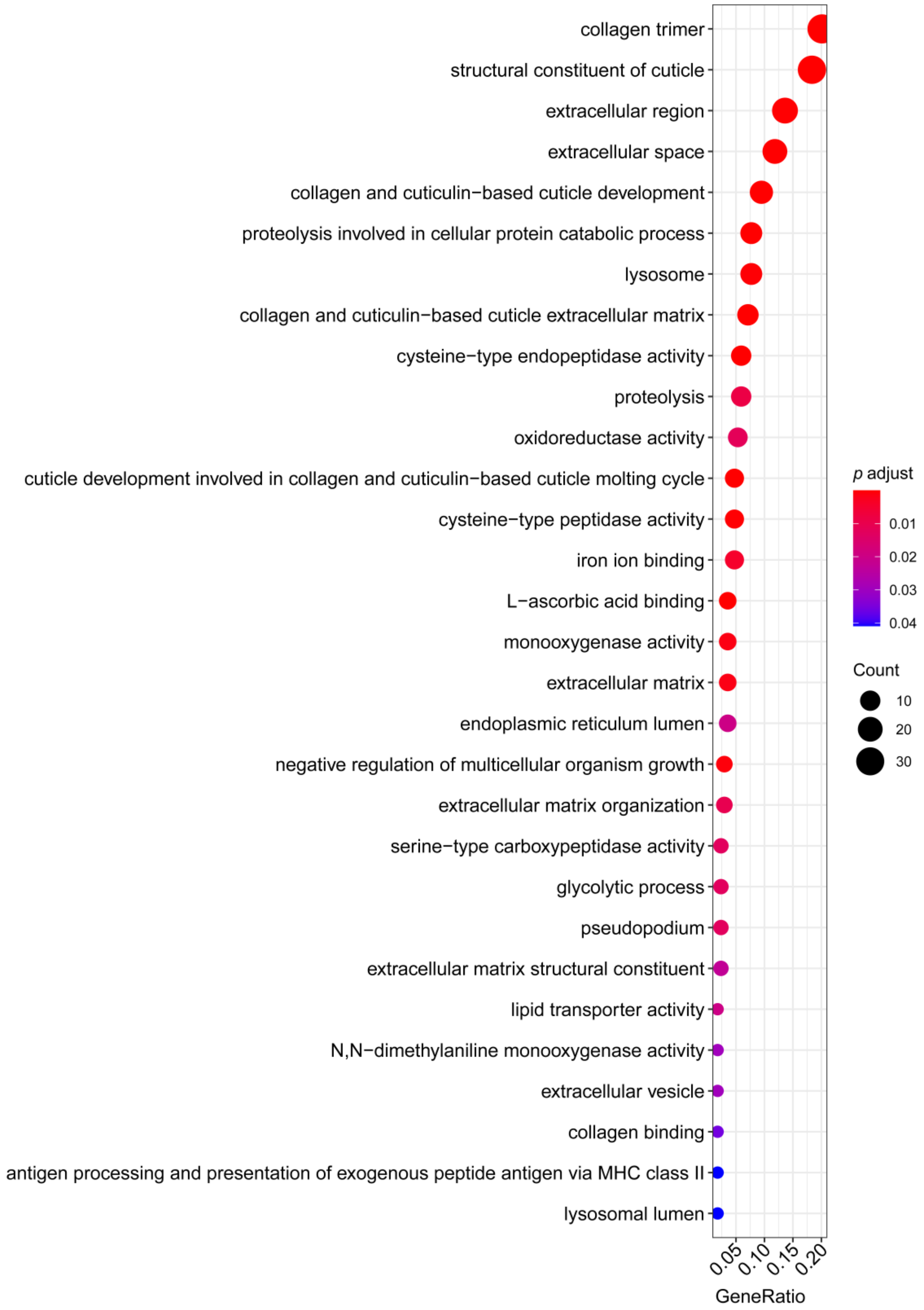

Figure S6 Distribution of GO function groups within up-regulated genes in PWNs treated by C18: 1, *p* adjust < 0.05.

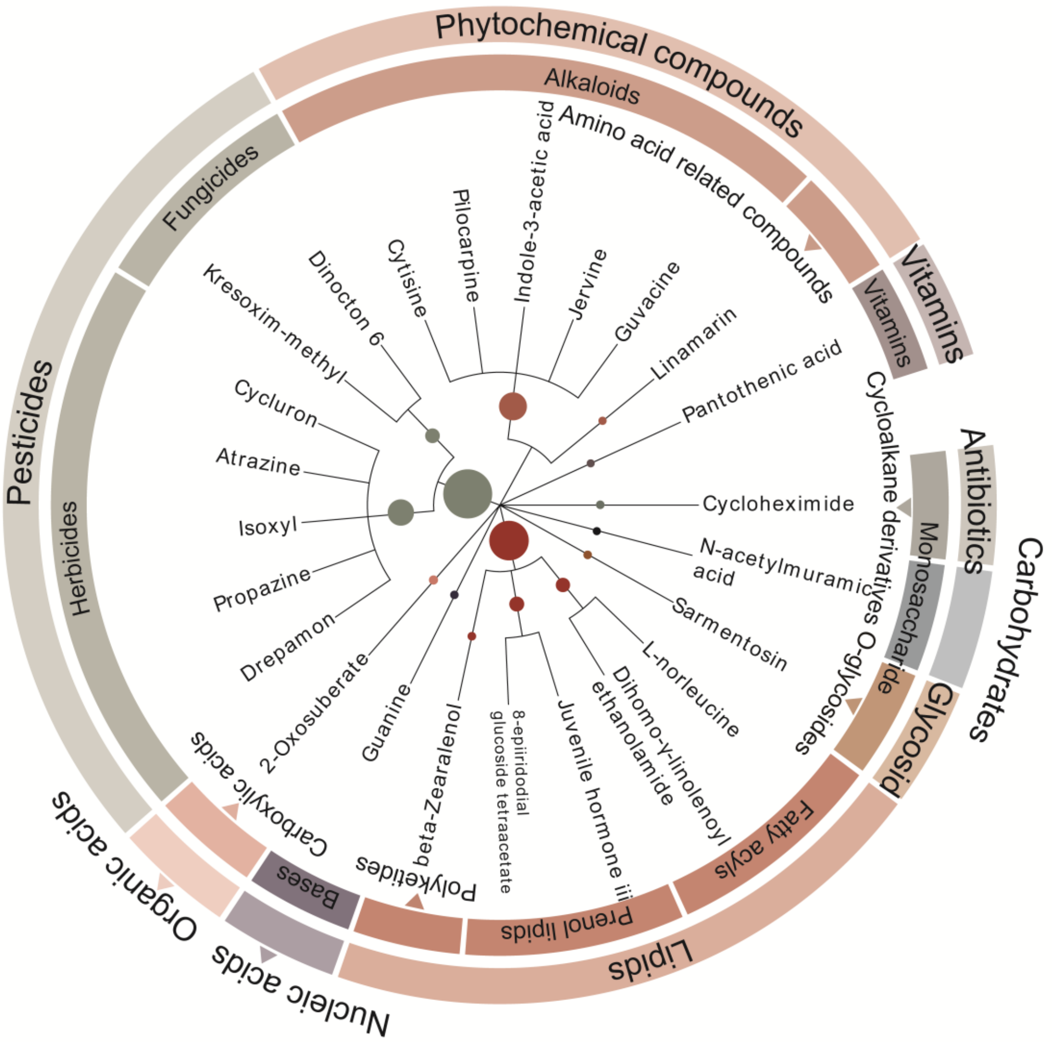

Figure S7 Enrichment analysis of up-regulated metabolites by *O. ips*.

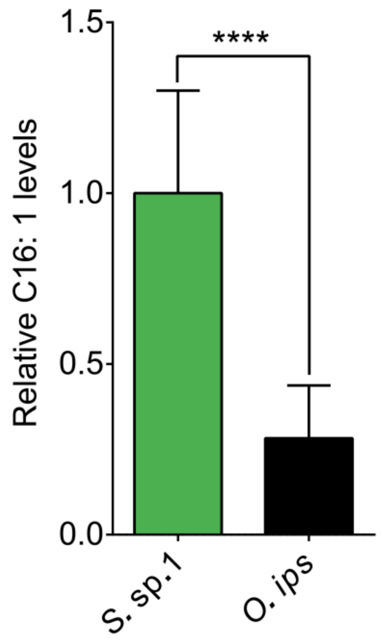

Figure S8 Relative C16: 1 levels of fungi cultured on xylem powder medium (*n*=6). ****, p < 0.0001; Student's *t* test.

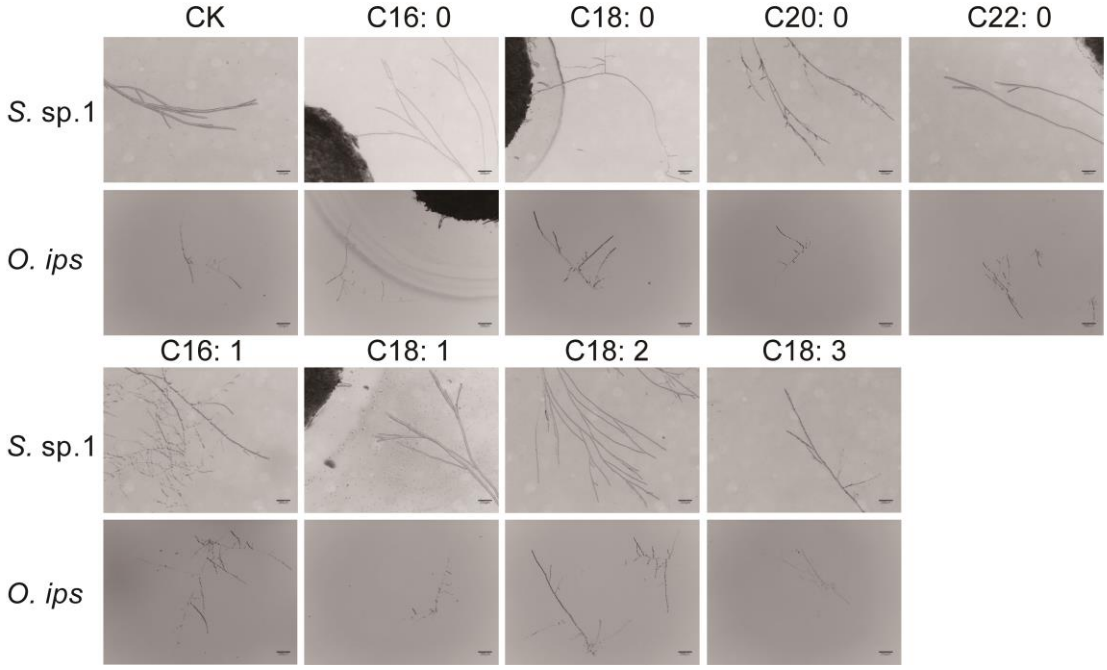

Figure S9 Induction of hyphal branching by different kinds of fatty acids.
